# A large-scale genome-wide gene expression analysis in peripheral blood identifies very few differentially expressed genes related to antidepressant treatment and response in patients with major depressive disorder

**DOI:** 10.1038/s41386-021-01002-9

**Published:** 2021-04-08

**Authors:** Anne Krogh Nøhr, Morten Lindow, Annika Forsingdal, Samuel Demharter, Troels Nielsen, Raimund Buller, Ida Moltke, Morana Vitezic, Anders Albrechtsen

**Affiliations:** 1 The Bioinformatics Centre, Department of Biology, University of Copenhagen, Copenhagen N, Denmark; 2 H. Lundbeck A/S, Valby, Copenhagen, Denmark

**Keywords:** Predictive markers, Depression

## Abstract

A better understanding of the biological factors underlying antidepressant treatment in patients with major depressive disorder (MDD) is needed. We perform gene expression analyses and explore sources of variability in peripheral blood related to antidepressant treatment and treatment response in patients suffering from recurrent MDD at baseline and after 8 weeks of treatment. The study includes 281 patients, which were randomized to 8 weeks of treatment with vortioxetine (*N* = 184) or placebo (*N* = 97). To our knowledge, this is the largest dataset including both gene expression in blood and placebo-controlled treatment response measured by a clinical scale in a randomized clinical trial. We identified three novel genes whose RNA expression levels at baseline and week 8 are significantly (FDR < 0.05) associated with treatment response after 8 weeks of treatment. Among these genes were *SOCS3* (FDR = 0.0039) and *PROK2* (FDR = 0.0028), which have previously both been linked to depression. Downregulation of these genes was associated with poorer treatment response. We did not identify any genes that were differentially expressed between placebo and vortioxetine groups at week 8 or between baseline and week 8 of treatment. Nor did we replicate any genes identified in previous peripheral blood gene expression studies examining treatment response. Analysis of genome-wide expression variability showed that type of treatment and treatment response explains very little of the variance, a median of <0.0001% and 0.05% in gene expression across all genes, respectively. Given the relatively large size of the study, the limited findings suggest that peripheral blood gene expression might not be the best approach to explore the biological factors underlying antidepressant treatment.

## Introduction

Major depressive disorder (MDD) is a commonly occurring mental disorder [1, 2]. Core symptoms of MDD listed by the Diagnostic and Statistical Manual IV (DSM-IV) include, sleep disturbance, dysphoria, anhedonia, and cognitive disabilities [3]. The disorder is often recurrent or even chronic, thus linked to reduced quality of life, medical morbidity, and mortality [4, 5]. In addition, MDD is a very heterogeneous disorder in terms of symptom profiles, comorbidities, and etiology as both environmental and genetic factors may contribute to the risk of MDD [6].

The most prescribed antidepressant medications for the treatment of MDD are second-generation antidepressants, including selective serotonin reuptake inhibitors (SSRIs) and serotonin norepinephrine reuptake inhibitors, which compared to first-generation antidepressants are safer and better tolerated [7]. Although second-generation antidepressants are generally beneficial in the treatment of MDD, 37% of patients do not respond during 6–12 weeks of treatment and 53% of patients do not achieve remission [7]. Individual response to a specific treatment can vary widely, and today limited evidence can guide the choice of one medication over another based on greater effectiveness and efficacy [7, 8]. Therefore, the selection of a first-line medication for a given patient is primarily the clinician’s subjective opinion. To better understand treatment response and to guide the choice of antidepressant medication for each individual patient, we need to learn more about the biological factors underlying antidepressant treatment response and the mechanism of action of antidepressants.

Gene expression analysis may be useful to investigate the biological changes during treatment at a given point in time. Due to the limited access to neuronal tissue, transcriptomic studies have focused on gene expression changes in peripheral blood, which have shown to be a useful proxy for the measure of changes in gene expression in the central nervous system [9]. Few studies have investigated gene expression in peripheral blood related to treatment response [10–15] and antidepressant treatment [13, 16]. However, the studies were not placebo controlled and they are smaller in sample size (*N* < 136) and limited in number of significant genes after adjustment for multiple testing.

The aim of this study is to investigate changes and sources of variability in gene expression assessed in peripheral blood related to antidepressant treatment and treatment response in patients suffering from recurrent MDD. To our knowledge, this is the largest dataset to date including both gene expression and placebo-controlled treatment response measured by a clinical scale in a randomized clinical trial. First, we identify differentially expressed genes between baseline and 8 weeks of treatment, and genes whose gene expression is correlated with treatment response at baseline and week 8. Then we test whether the expression of the significant genes in these tests depend on the type of treatment (placebo vs. vortioxetine). Next, we compare our findings to current literature. Finally, we investigate what amount of genome-wide expression variance is explained by the severity of depression at baseline, type of treatment, treatment response and other variables relevant for depression.

## Materials and methods

### Patients and study design

This study included a subset of 281 European genetic ancestry, depressed patients (age ≥18 and ≤65 years) from a randomized, double-blind, placebo-controlled study evaluating the efficacy of acute treatment with 10 and 20 mg/day vortioxetine vs. placebo on cognitive dysfunction. First, the patients went through a screening period of up to 10 days. At baseline, the patients were randomized (1:1:1) to 8 weeks of double-blind treatment of vortioxetine 10 mg/day, vortioxetine 20 mg/day, or placebo. Patients in the vortioxetine 20 mg/day group received a dose of 10 mg/day during week 1 and from weeks 2 to 8 they received 20 mg/day.

The patients included in this study suffered from recurrent MDD diagnosed using DSM-IV-TR [3]. They were currently in a depressive episode of ≥3 months’ duration (confirmed using the Mini International Neuropsychiatric Interview [17]) and had a Montgomery-Åsberg Depression Rating Scale (MADRS) total score ≥26 at screening and baseline visits. The exclusion criteria are described in (McIntyre, Lophaven, and Olsen 2014) and include: Any current Axis I disorder other than MDD, significant risk of suicidality, taking psychotropic drugs within 2 weeks prior to baseline or during the study. The patients were from 66 outpatient and inpatient settings from December 2011 to May 2013 located in 12 different countries: Australia (*N* = 2), Canada(*N* = 7), Finland (*N* = 59), France (*N* = 9), Germany (*N* = 26), Latvia (*N* = 21), Mexico (*N* = 34), South Africa (*N* = 3), Serbia (*N* = 11), Slovakia (*N* = 33), Ukraine (*N* = 33), and the USA (*N* = 43).

Prior to the study, local research ethics committees approved the design of the trail and patients signed a general informed consent form (ICF) and a separate ICF for exploratory genetic research purposes. The approval for analysis of RNA was obtained from the Danish National Ethics committee under number: 1802757 (title: Predicting the response of medical treatment of patients with depression: An analysis of RNA and microRNA from blood samples collected in the clinical study 14122A.).

MADRS total score was used to assess symptom severity of depression. Change from baseline in MADRS total score after 8 weeks treatment with vortioxetine or placebo will be referred to as treatment response.

### RNA extraction and sequencing

Blood samples were collected in PAX gene tubes at both baseline and week 8 resulting in a total of 562 samples. The samples were stored by the Lundbeck Biobank located at BioStorage Technologies Inc., Indianapolis, Indiana, United States. For RNA extraction, the samples were sent directly from the Lundbeck Biobank to QIAGEN Genomic Services Enterprise labs in Hilden, Germany. Subsequently, the extracted RNA was sequenced by QIAGEN, Maryland, United States. QIAGEN performed all the RNA processing and sequencing steps according to their internal standards.

In short, RNA was extracted from whole blood and rRNA depletion and globin mRNA depletion were performed. Upon quality control, only samples with a RIN value above 7.0 were kept. After whole transcriptome library building and quality control, the sequencing was performed on Illumina machines with double stranded 2 × 50 bp protocol and 60 M raw reads per sample. FASTQC [18] was performed to check the samples quality. Samples were then mapped to Genome Reference Consortium Human Build 38 patch release 12 using STAR [19] and quantified using Stringtie [20]. Only uniquely mapping sequences were kept. For annotation GRCh38 GENCODE v28 [21] was used. Step by step command lines are available in Supplementary Material 1A.

### Gene expression analysis

We performed gene expression analyses to identify differentially expressed genes related to antidepressant treatment and treatment response. Thus, two gene expression analyses were conducted:

Test 1: Genes differentially expressed between baseline and 8 weeks of treatment

Test 2: Genes whose expressions are correlated to treatment response at baseline and week 8.

The tests were conducted using the R package Dream [22], as both analyses contain repeated measures, and Dream can account for these by fitting linear mixed models with a random effect for each patient. More specifically, *y*_*g*_ is a vector containing counts per million for gene *g*. *x*_*i*_ is the *i*th covariate in the experimental design with coefficients $\beta _i^g$. The first nine covariates are the same for both tests (PEER factor 1—PEER factor 7, age, sex). The tests also include a covariate and coefficient for type of treatment (*x*_*treat*_, *β*_*treat*_), symptom severity at baseline (*x*_*MA BL*_, *β*_*MA BL*_), and treatment response (*x*_*TR*_, *β*_*TR*_). *Subject* is a random effect with gaussian coefficients *α*_*g*_ with variance ... *ε*_*g*_ are errors modeled with precision weights *w*_*g*_. In test 1 moderated t-statistics using an empirical Bayes approach is applied to test if the difference between baseline and week 8 (*β*_*visit*_) is equal to zero for each gene. In test 2, a similar approach is used to test if treatment response (*β*_*TR*_) is constant for each gene. Therefore, test 1 and 2 using DREAM will be:

$$Test\,1\!\!:\,log2\,y_g =	 \, \mathop {\sum }\limits_{i = 1}^9 x_i\beta _i^g \,+\, x_{treat}\beta _{treat}^g \,+\, {\boldsymbol{{x}}}_{\boldsymbol{{visit}}}{\mathbf{\beta}} _{\boldsymbol{{visit}}}^{\boldsymbol{g}} \\ 	\, +\,subject\,\alpha _g \,+\, \varepsilon _g\\ Test\,2\!\!:log2\,y_g =	 \, \mathop {\sum }\limits_{i = 1}^9 x_i\beta _i^g \,+\, x_{treat}\beta _{treat}^g \,+\, x_{visit}\beta _{visit}^g \,+\, x_{MA\,BL}\beta _{MA\,BL}^g \,+\, {{\boldsymbol{{x}}}_{\boldsymbol{TR}}}{\mathbf{\beta}} _{\boldsymbol{{TR}}}^{\boldsymbol{{g}}} \\ 	 +\, subject\,\alpha _g \,+\, \varepsilon _g$$

where $$\varepsilon _g \sim N\left( {0,diag\left( {w_g} \right)\sigma _\varepsilon ^2} \right).$$

Following both tests, we explored if the gene expression of the significant genes depended on the type of treatment, placebo, or vortioxetine, by including an interaction term in test 1 between visit and type of treatment, and in test 2 between treatment response and type of treatment.

In addition, we conducted three exploratory gene expression analyses that do use random effects using Deseq2:

Test 3: Genes differentially expressed between placebo and vortioxetine at week 8.

Test 4: Genes whose expressions are correlated to treatment response at baseline.

Test 5: Gene whose expressions are correlated to treatment response at week 8.

A detailed description of these tests is given in Supplementary Material 1B. Furthermore, various methods can be used to conduct gene expression analysis, see Supplementary Material 1C for more information on our rationale behind choosing the methods.

Identification and removal of unknown sources of variation in gene expression, such as multi-site effects and batch effects, are strongly recommended and can improve RNA-seq studies significantly [23]. In this study, the method PEER [24] was used. PEER uses a Bayesian approach to calculate hidden factors, called PEER factors. The PEER factors can be used as covariates in differential gene expression analysis to account for unknown sources of variation that is not explained by the covariates of interest. In the current study, PEER factors were calculated using all samples and accounting for all covariates of interest in the experimental design. In the gene expression analyses, the first seven PEER factors were included as covariates, for an explanation of why seven PEER factors were chosen, see Supplementary Material 1D. Sex and age were also included as covariates in the gene expression analyses. Furthermore, when testing for genes associated with treatment response the MADRS total score at baseline was included as a covariate.

We perform five gene expression analyses where each analysis corrects for multiple testing using Benjamini–Hochberg false discovery rate (FDR). To account for the five test settings, we only consider genes significant if they have a FDR < 0.01. Furthermore, in Test 1, which has a categorical predictor, genes are not considered significant if they have an absolute Log2(FC) < 0.07 (corresponding to a FC between 1.05 and 0.95). In Test 2, which has a continuous predictor, genes are not considered if they have an absolute Log2(FC) < 0.005 (corresponding to a FC of 0.0016 for each point on the continuous scale). All gene expression tests are provided in Supplementary Material 2. Note, the samples from patients treated with 10 mg vortioxetine and 20 mg were pooled in all analyses to increase power since responses of the two groups were similar.

### Comparison with findings in current literature

The current literature was reviewed to find genes already identified as significantly related to antidepressant treatment and treatment response. Studies with *N* < 20 were not considered. To our knowledge, no genes with a significant association with a specific antidepressant treatment have been discovered up to now. However, ten genes have been significantly (FDR < 0.05) associated with treatment response and were highlighted as interesting in previous literature: *SMAD7* [10], *SIGLECP3* [10], *MMP28* [15], *KXD1* [15], *IRF7* [11], *NR2C2* [14], *ZNF641* [14], *YWHAZ* [14]*, NLGN2* [14], and *FKBP1A* [14]. We investigated whether any of these findings could be replicated in our study. This was done for each gene by performing a test similar to the test in which the gene was found to be significant. The majority of the genes were identified in tests comparing responders to non-responders. To enable this comparison, we defined responders as patients with a >50% reduction from baseline in the MADRS total Scale.

### Analysis of variance

The amount of variance explained by variables relevant for depression and the subsequent analysis across all samples was explored and quantified using the R package variancePartition [25]. The method fits a linear mixed model to each gene and the total variance is partitioned to the variables of interest and the residual variance. The variance is normalized to sum to 1 for each gene [25]. The variance of a variable for a gene is reported as a percentage after correcting for all other variables included in that analysis.

## Results

### Patient characteristics

This study includes 281 patients of European genetic ancestry. An overview of demographics and baseline assessments is shown in Supplementary material 1 Table 5. There was no significant difference in demographics (gender, age, length of current major depressive episode (MDE), and no. of previous MDE) or baseline assessment of severity of depression between placebo and vortioxetine-treated patients using t-tests (unequal variance for the two groups is assumed) and Fisher’s exact test.

### Efficacy

The average MADRS total score was 31.89 at baseline. After 8 weeks of treatment, the change in MADRS total score was −12.62 (SD = 10.44) for placebo, −16.43 (SD = 9.56) for vortioxetine 10 mg and, −18.48 (SD = 8.60) for vortioxetine 20 mg. Both vortioxetine 10 mg and vortioxetine 20 mg showed a greater improvement compared to placebo at week 8 using *t*-tests (unequal variance for the two groups is assumed). The difference in means of treatment response at week 8 between placebo and vortioxetine 10 mg was −3.82 (*t*(186.77) = 2.62, *p* value = 0.0095) and the difference was −5.86 between placebo and vortioxetine 20 mg (*t*(183.42) = 4.22, *p* value < 0.0001).

### Genes differentially expressed between baseline and week 8

Comparison of gene expression at baseline to gene expression after 8 weeks of treatment revealed no significant genes. However, seven genes had a *p* value below 0.001, see Supplementary Material 2 Table 2, which could be interesting for future studies.

### Gene expression related to treatment response

Change in gene expression related to treatment response was investigated at baseline and week 8 for both treatment types (placebo and vortioxetine), see Supplementary Material 2 Table 5. Genes with an FDR < 0.05 are presented in Table 1. It was also explored if the significant changes in gene expression were associated with depression symptom severity at baseline.

**Table 1:** Genes with differential expression correlated to treatment response.

Gene	Mean	Log2FC (SE)	FDR	PVE (%)	*p* value MADRS BL	*p* value treatment
Baseline and week 8						
*PROK2*	*146.54*	*−0.014 (0.0025)*	*0.0028*	*2.38*	*0.88*	*1.00*
*SOCS3*	*76.70*	*−0.016 (0.0031)*	*0.0039*	*5.52*	*0.93*	*0.11*
*GCA*	*663.43*	*−0.006 (0.0012)*	*0.0075*	*0.58*	*0.65*	*0.43*
*GALNT14*	7.61	−0.017 (0.0035)	0.015	3.07	0.67	0.72
*UBE2J1*	210.26	−0.008 (0.0017)	0.017	2.13	0.59	0.025
*GNG10*	48.91	−0.011 (0.0025)	0.017	0.49	0.15	0.96
*FRAT2*	286.38	−0.0057 (0.0013)	0.022	1.31	0.85	0.62
*ADK*	39.45	−0.0084 (0.0019)	0.024	2.81	0.049	0.85
*PSG4*	2.55	−0.027 (0.0063)	0.042	3.19	0.93	0.72

Mean = mean of counts per million using DREAM; Log2FC (SE) = log2 fold change (standard error); FDR = BH adjusted *p* value; PVE = Proportion of Variance in gene expression Explained by treatment response; *p* value MADRS BL = *p* value of association between gene expression and depression symptom severity at baseline; *p* value treatment = *p* value of the interaction effect between treatment response and treatment type. Significant genes are in italics.

Investigation of gene expression at both baseline and week 8 related to treatment response identified 47 genes with a *p* value < 0.001. Nine genes had an FDR < 0.05, of which three genes, prokineticin 2 (*PROK2*), suppressor of cytokine signaling-3 (*SOCS3*), and *GCA*, were significantly related to treatment response at both time points. None of the genes showed an interaction between treatment response and type of treatment.

We also explored if gene expression was related to treatment response at baseline or week 8. Only at baseline, we identified 1 gene with an FDR < 0.1, which was related to treatment response, see Supplementary Material 1A. Downregulation of *PROK2* (FDR = 0.0016) significantly predicted a poorer treatment response regardless of treatment type.

Since none of the significant genes depended on whether the treatment was vortioxetine or placebo, we explored if there were any genes differentially expressed between placebo and vortioxetine after 8 weeks of treatment. In this analysis, we did not find any significant genes, see Supplementary Material 1A.

### Examination of differentially expressed genes correlated to treatment response

**Fig. 1:**
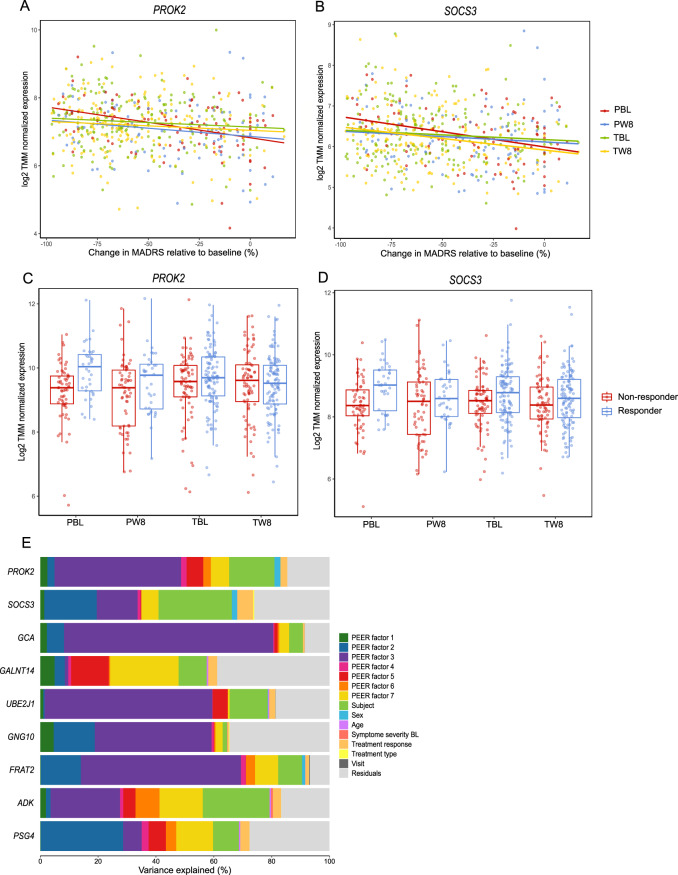
Examination of genes correlated to treatment response. Plots of log2-transformed TMM-normalized gene expression as a function of percentage improvement in MADRAS total score at week 8 from baseline for *PROK2* (**A**) and *SOCS3* (**B**). Percentage improvement is plotted to account for the MADRS score at baseline, since change in MADRS score depends on the MADRS score at baseline. Boxplots of log2-transformed TMM-normalized gene expression difference between responders and non-responders for each timepoint (baseline and week 8) and each treatment type (placebo and vortioxetine) for *PROK2* (**C**) and *SOCS3* (**D**). PBL = samples from placebo-treated patients at baseline; PW8 = samples from placebo-treated patients at week 8; TBL = samples from vortioxetine-treated patients at baseline; TW8 = samples from vortioxetine-treated patients at week 8. Response is defined as >50% decrease in the MADRS total score from baseline. **E** Bar plot of variance partitioned on the covariates in the experimental design for the nine genes, with an FDR < 0.05, related to treatment response.

*PROK2* (FDR = *0.0028*) and *SOCS3* (FDR = *0.0039*) were the most significant genes with changes in expression related to treatment response at both baseline and week 8. Downregulation of both genes was related to a poorer treatment response. Figure 1A–D shows TMM-normalized gene expression of both genes as boxplots for responders and non-responders and as a function of percentage improvement in MADRAS total score at week 8 from baseline for both genes.

Sources of variability in gene expression of the nine genes in Table 1 were investigated, see Fig. 1E and Supplementary Material 2 Table 6. The main drivers of variance of *SOCS3* expression were the PEER factors (sum to 40.85%), subject (25.35%), sex (1.86%), and treatment response (5.52%). Even without the PEER factors explained treatment response 2.71%. For *PROK2* were the main drivers of variance also the PEER factors (sum to 65.30%), subject (15.67%), sex (2.04%), and treatment response (2.38%). Without the PEER factors, treatment response explains 2.30%.

The amount of variability in treatment response due to expression of the genes in Table 1 was also explored, see Supplementary Material 2 Table 6. TMM-normalized and log2-transformed gene expression of *SOCS3* explained 2.44% of variation in treatment response, after adjustment of MADRS score at baseline, age, and sex. In comparison, the MADRS score at baseline explains 9.83% of the variance. After 8 weeks of treatment, the variance in treatment response was explained by *SOCS3* expression 2.94%. *PROK2* expression explained at baseline 3.05% of the variation in treatment response and at week 8 1.80%.

### Genes overlapping with current literature

None of the ten genes significantly associated with treatment response in the current literature was replicated in our study, see Table 2. However, the direction of *NR2C2*, *ZNF641, FKBP1A, SMAD7*, and *KXD1* were the same in the current literature compared to our study. In this study, the genes *NLGN2*, *SIGLECP3*, *MMP28*, and *IRF7* were lowly expressed and were therefore not tested.

**Table 2:** Genes with FDR < 0.05 and highlighted as interesting in previous literature.

	Previous studies			Our study				
Gene	N	Test	Direction	Test	Average exp	Effect size (SE)	*p* value	Direction
*YWHAZ* [14]	87	R vs. NR (W4)	R↑	TR (W8)	3607.28	0.0014 (0.0015)	0.3509	TR↓
*NLGN2* [14]	84	R vs. NR (W12)	R↓	TR (W8)	0	–	–	–
*NLGN2* [14]	61	R vs. NR (BL)	R↓	TR (BL)	0	–	–	–
*NR2C2* [14]	84	R vs. NR (W12)	R↓	TR (W8)	536.27	0.0019 (0.0028)	0.4814	TR↓
*ZNF641* [14]	84	R vs. NR (W12)	R↓	TR (W8)	423.28	0.00077 (0.0026)	0.7648	TR↓
*FKBP1A* [14]	84	R vs. NR (W12)	R↑	TR (W8)	433.95	−0.0024 (0.0021)	0.2541	TR↑
*SMAD7* [10]	77	R vs. NR (BL)	R↓	TR (BL)	110.28	0.0012 (0.0028)	0.6640	TR↓
*SIGLECP3* [10]	77	R vs. NR (BL)	R↓	TR (BL)	23.93	–	–	–
*MMP28* [15]	39	TR (W8)	TR↑	TR (W8)	0.60	–	–	–
*KXD1* [15]	39	TR (W8)	TR↓	TR (W8)	89.01	0.0030 (0.0029)	0.3003	TR↓
*IRF7* [11]	63	TR (BL)	TR↑	TR (BL)	0.69	–	–	–

R = responders or remitters definitions are different between studies; TR↑ = increased expression result in greater response; R↑ = responders have higher expression compared to NR; – = have less than six counts for more than 200 patients.

*NR* non-responders, *TR* treatment response (continious varibale).

### Genome-wide variance

**Fig. 2:**
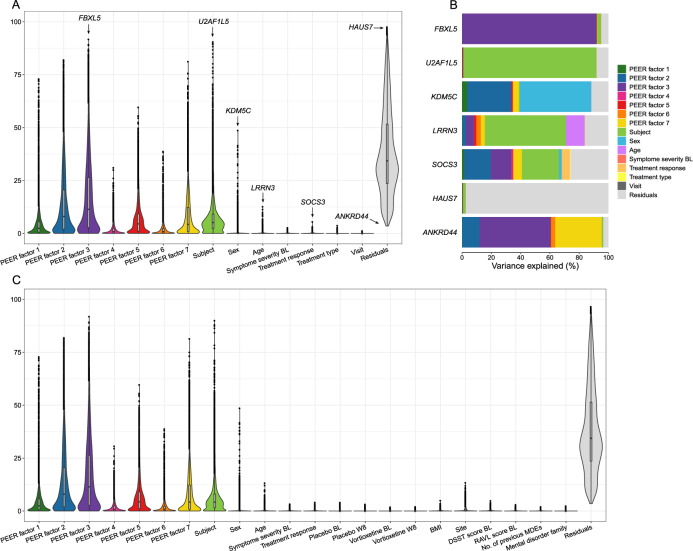
Examination of the distribution of variance across all genes. **A** Violin plot of the distribution of variance across all genes for the covariates in the experimental design. Inside the violin plots are boxplots. **B** Bar plot of variance partitioned on the covariates in the experimental design for the genes highlighted in plot A. The variance of the covariates for each gene sum to 1. **C** Violin plot of the distribution of variance across all genes for the covariates in the experimental design and explorative variables.

Although this study is the largest gene expression study in blood with a placebo-controlled treatment response measured by a clinical scale in a randomized clinical trial to date, only a few genes were significant after multiple testing and no genes identified in current literature were replicated. Therefore, the distribution of variance across all genes was investigated for each covariate in the experimental design, see Fig. 2A and Table 3.

**Table 3:** Parameters describing variance across all genes for each covariate in the experimental design.

Covariates	Median variance (%)	Mean variance (%)	No. of genes >5%	No. of genes >2%	No. of genes >1%	Max variance (%)
Sex	2.51 × 10^−7^	0.34	184	419	875	48.63
Age	0.07	0.22	23	159	573	12.70
Visit	5.33 × 10^−8^	0.03	0	0	14	1.22
Treatment type	3.92 × 10^−8^	0.03	0	5	44	3.74
Symptom severity at baseline	0.05	0.12	0	7	92	2.71
Treatment response	0.06	0.18	1	38	388	5.52
PEER factor 1	2.40	4.32	3808	7274	9134	73.02
PEER factor 2	8.00	13.33	8094	10096	11099	82.02
PEER factor 3	11.37	16.93	8896	10632	11405	91.74
PEER factor 4	0.87	1.73	1147	3871	6142	31.07
PEER factor 5	4.52	6.83	6333	8991	10239	59.64
PEER factor 6	0.87	1.66	731	3845	6247	38.76
PEER factor 7	4.28	8.24	6249	8481	9771	81.23
Subject	5.13	7.69	6864	10432	11452	90.55
Residuals	34.32	38.34	13379	13416	13416	97.56

Median variance = median variance across all genes; Mean variance = mean variance across all genes; No. of genes > X% = No. of genes for which the covariate explains more than X% of the variance. Max variance = variance of the gene for which the covariate explains the most variance.

The PEER factors captured a total median of 32.26% of the variability genome-wide. Variance across subjects explained a median of 5.13%. Note, variance across subjects depends on nested variables, such as sex. Removing sex as a covariate increases the variance explained by subjects from 5.13 to 5.31%, since sex and other nested variables are properties of subject and not sample. Each subject has two samples and sex is the same for both samples.

The remaining covariates (age, sex, visit, treatment type, depression symptom severity at baseline, treatment response) in the experimental design explained less than a total median of 1% of the total variance. Sex explained a median variance of 2.51 × 10^−7^ and for 184 genes sex explained more than 5% of the variance, out of which 175 genes were present on the X or Y chromosome. This indicates that sex explains little variability across all genes but has a strong effect on a small number of genes. The median variance explained by depression symptom severity at baseline (0.05%) and treatment response (0.06%) were higher compared to the median variance explained by sex. For only 7 and 38 genes did depression symptom severity at baseline and treatment response, respectively, explain more than 2% of the variance in gene expression. Type of treatment (3.92 × 10^−8^%) and visit (5.33 × 10^−8^%) had low median variance and the number of genes for which type of treatment visit explained more than 1% was only 44 and 14 genes, respectively. This shows that very little of the variance in gene expression is due to variables related to depression.

After accounting for the variance explained by all covariates in the experimental design, the median amount of unexplained variation, the residuals, was 34.32%. Excluding the PEER factors from the analysis increase the median variance explained by the residuals to 91.19%.

The distribution of variance across all genes was also investigated for a set of explorative variables (including DSST score at BL, Rey Auditory Verbal Learning score at BL, no. of previous MDEs, mental disorder family, site, and BMI) combined with the covariates in the experimental design, see Fig. 2C. The explorative variables all explained a low (<0.00072%) median variance and all variables except from site did not explain more than 5% of variance in gene expression for any genes. After including the explorative variables, the distribution of the experimental variables remained almost identical. The biggest difference was observed for the median variance across subjects, which were reduced from 5.13 to 4.23% when including the explorative variables. This reduction was expected, since all the explorative variables are properties of subject (nested variables).

Spearman correlation was investigated for all pairs of the explorative variables and the covariates in the experimental design, see Supplementary Material 1 Fig. 6. Subject and site were correlated with all the covariates. The first seven principal components (PC) were also investigated. As expected, the PCs are not correlated with each other, but they are correlated with the PEER factors.

## Discussion

Here we present the largest study to date using next-generation sequencing to assess gene expression changes in SSRI treatment and treatment response in peripheral blood in patients from a randomized clinical trial suffering from MDD. While similar studies have been done before, they were focusing on different treatments [10–16], technologies [10–15], and did not have a placebo group [10–16]. Despite the bigger size of our study, we did not identify any significant genes after multiple test correction in either the comparison between the two time points or treatment and placebo.

Our analysis of treatment response identified a few significant differentially expressed genes (FDR < 0.01); one gene (*PROK2*) was predictive of treatment response at baseline and three genes (see Table 1) were related to treatment response at both baseline and week 8. Interestingly, we did not replicate any of the genes previously reported. Examination of variance in gene expression genome-wide suggests that a small amount of the variance in gene expression in whole blood is due to type of treatment or treatment response.

Among the genes significantly related to treatment response at both baseline and week 8 were *PROK2* and *SOCS3*. They were both associated with poorer treatment response at baseline and after 8 weeks of treatment. *PROK2* is linked to circadian clock regulation [26] which in turn is associated with multiple mood disorders, such as depression, bipolar disorders, and seasonal affective disorder [27]. One previous study has identified it as one of the seven genes whose upregulation is predictive of MDD disease status using blood expression profiles [28]. In our study, there was no association of *PROK2* with the severity of depression. However, downregulation of *PROK2* was significantly associated with poorer treatment response, explaining as much as 3.05% variation in treatment response at baseline and 1.80% variation at week 8. Expression of *PROK2* has never been linked to treatment response in depression before.

The other significant gene, *SOCS3* is a major regulator of inflammation and infection [29]. Several studies are implicating a role of inflammation in MDD [30]. While we can only speculate on the direct involvement of *SOCS3*, it is a negative regulator of hormone and cytokine signaling by regulating the JAK-STAT pathway. A recent mice study reported no difference in expression of *SOCS3* between depressed and healthy mice. However, the study did observe a significant increase in *SOCS3* expression when the mice were treated with an antidepressant [31]. The *STAT3/SOCS3* pathway has been suggested to be involved in the pathogenesis of depression [31].

Previous studies that have investigated treatment response [10–15] have so far identified and highlighted a total of ten genes related to treatment response [10, 11, 14, 15]. In the current study, we identified one gene significantly predictive of treatment response at baseline and three genes related to treatment response at both baseline and week 8. To date, no significant genes have been replicated in gene expression studies. Nor did we replicate any of the previously identified genes. This could be because treatment response has been studied for various antidepressant medications and the different medications result in the expression of different genes. Another possible reason could be that treatment responses have a small effect on a large number of genes, which was exactly what we observed in our analysis of variance transcriptome wide. If this is the case, the sample sizes of previous studies (*n* = 52–139) and our study (*n* = 281) may not be large enough to detect all the genes in the large pool of genes with small effect sizes. Finally, it is possible that some of the genes identified in previous studies and in our study are false positives.

The most interesting and novel question in this study is the ability to differentiate between antidepressant treatment and placebo. Previous studies did not include a placebo group. Consequently, it is unknown if the gene expression changes were effects of the antidepressant treatments or a placebo effect. In our study, the findings were limited when comparing placebo to treatment. We did not identify any significant genes between placebo and antidepressant treatment at week 8 and the significant genes related to treatment response were also independent of treatment type. Furthermore, we did not identify any significant genes between baseline and post treatment. Similarly, previous studies [13, 16] have not identified any significant genes after antidepressant treatment. It is likely that an even larger sample size is needed to detect differentially expressed genes related to changes after antidepressant treatment and to separate placebo from an antidepressant treatment. This is supported by our investigation of variance in gene expression, which showed that type of treatment (placebo or vortioxetine) and visit (baseline and week 8) explained little variation transcriptome wide and had a small effect. Due to the small effect of type of treatment and visit it is possible that the true positives are drowning in false positives.

A possible reason for our limited findings both when investigated treatment response and antidepressant treatment could be the heterogeneity among patients. There are various sources of heterogeneity in MDD. Some of the sources of heterogeneity in our sample which we did not integrate in our analyses include different symptom profiles, age of MDD onset, group characteristics (genetic ancestry, culture ect.), comorbidities, and etiology (genetic and environmental factors). Another possible reason could be, that the effect of and response to antidepressants are not reflected in peripheral blood gene expression. Peripheral blood has been a widely used tissue to evaluate neuropsychiatric disorders since it is easy to access. A review of transcriptomic studies showed that between 35 and 80% of known transcripts are present in both blood and brain tissue samples. Furthermore, the review indicated that cross‐tissue correlation in expression levels range from 0.25 to 0.64 [9]. This suggests that gene expression from peripheral blood could be useful for examining the effect of antidepressants in patients suffering from MDD. However, our study indicates that peripheral blood gene expression might not be the best approach. Therefore, when using peripheral blood for evaluation of treatment response and antidepressant treatment other omics data might be more successful or other tissues could be considered.

Even though none have passed multiple test correction, several interesting genes were differentiated between baseline and week 8. Among these are *FKBP5* and *Noggin* (*NOG*), which were upregulated and downregulated at week 8, respectively. Variants of *FKBP5* have been associated with regulation of the hypothalamus‐pituitary‐adrenocortical axis [32], MDD risk [33, 34], and antidepressant treatment response [32, 35]. *NOG* overexpression has shown increased hippocampal neurogenesis and reduced depression and anxiety-like behaviors [36].

In the current study, we examined the genome-wide distribution of transcription variance explained by variables relevant for depression in a clinical study. Gene expression have multiple sources of biological and technical variation. The current literature has not yet explored how much variance across genes is explained by psychiatric phenotypes like severity of depression, treatment, and response to treatment. Exploration of variation in gene expression will give a better understanding of how much gene expression can be used to learn about MDD. We learned that depression symptom severity at baseline and treatment response have a small effect on many genes compared to e.g., sex, which in our study showed a large effect on a small number of genes. For type of treatment (placebo and vortioxetine) and visit (baseline and week 8), our analysis indicated that these variables explain both little variation genome-wide and have small effect sizes. Examining outcome variables with a small effect on gene expression in a gene expression analysis with thousands of genes is likely to have a high proportion of false positives. Consequently, sample size and removal of unknown sources of variation becomes crucial. In our study, we applied the method PEER to correction of unknown sources of variation. We used the method to calculate seven PEER factors, which captured a total median of 32.26% of the variation in gene expression across all genes. It was beyond the scope of this paper to investigate which sources of variation the PEER factors remove. A study evaluating different methods used to remove variation in RNA-seq data showed, that PEER [24] removed the majority of false positives, were best at removing site-specific bias and removed variance significantly associated with GC-content, gene body coverage uniformity, and average base error rate and insert size [23]. We also observed great advantage in using this method, and we strongly recommend using this method or a similar method in RNA-seq studies, especially when working with outcome variables with little effect on gene expression.

In summary, this study demonstrates that it is feasible to identify transcriptome-wide significant gene expression changes associated with antidepressant response, although the amount of variation in gene expression explained by treatment response is small. In parallel to transcriptomic analyses of antidepressant response, genome-wide association studies (GWAS) are emerging. The best powered GWAS of antidepressant response demonstrates that common genetic variation contributes to treatment response, although no genome-wide significant loci have been identified [37]. Further there is a genetic correlation between antidepressant response and educational attainment and schizophrenia [37]. Collectively, large-scale omics analyses are beginning to deliver insight to the underlying biology of treatment response to antidepressants in MDD.

## Supplementary information

Supplementary Material 1

Supplementary Material 2
