# Supplementary material for: A large-scale genome-wide gene expression analysis in peripheral blood identifies very few differentially expressed genes related to antidepressant treatment and response in patients with major depressive disorder
Source: Neuropsychopharmacology. 2021 Apr 8;46(7):1324–32. doi: 10.1038/s41386-021-01002-9 (PMC8134553; doi:10.1038/s41386-021-01002-9)
Supplement: Supplementary file 1 — Supplementary Material 1 [file 41386_2021_1002_MOESM1_ESM.docx]

**SUPPLEMENTARY MATERIAL 1**

**A: Bioinformatics command line steps for alignment and annotation**

The following command lines were run on all the samples presented in this paper:

1. STAR command line:

./STAR --runThreadN 8 --genomeDir GRCh38_gencode_v28/star_index --readFilesIn FASTQ/ID1.R1.fastq.gz FASTQ/ID1.R2.fastq.gz --readFilesCommand zcat --outFileNamePrefix star/ --outReadsUnmapped Fastx --outSAMtype BAM SortedByCoordinate --outWigType bedGraph

2. Only keep unique mappings and remove scaffolding genes:

(Python code)

chromosome_regex = re.compile(r'^chr(?:\d+|X|Y)$', re.I)

def output_clean_bam(input_file, output_file):

"""

Remove multimapped matches from bam-file as well as chromosomes

not matching chromosomes_regex.

Output the to the resulting bam file: output_file

"""

with pysam.AlignmentFile(input_file, 'rb') as in_bam:

with pysam.AlignmentFile(output_file, 'wb', template=in_bam) as out_bam:

for line in in_bam:

# Only pick unique mappings

if line.mapping_quality == 255:

if not chromosomes_regex or chromosomes_regex.match(line.reference_name):

out_bam.write(line)

output_clean_bam(

'star/Aligned.sortedByCoord.out.bam',

'star/Filtered.bam')

3. Stringtie command line:

./stringtie -v -B star/Filtered.bam -G gencode.v28.chr_patch_hapl_scaff.annotation.gtf -p 4 -o filtered/filtered.gff

**B: Secondary gene expression analyses**

Three exploratory gene expression analyses were conducted:

Test 3: Genes differentially expressed between placebo and vortioxetine at week 8.
Test 4: Genes whose expressions are correlated to treatment response at baseline.
Test 5: Gene whose expressions are correlated to treatment response at week 8

Test 3, 4, and 5 were performed by fitting generalized linear models with negative binominal error distributions using the R package DESeq2 [23]. More specifically, we assume the count vector, $K_{g}$, for gene *g* follows a negative binomial distribution with mean $\mu_{g}$, dispersion $a_{g}$. The mean is composed of the quantity $q_{g}$ and normalization constant $s_{g}$. $x_{i}$ is the *i*th covariate in the experimental design with coefficients $\beta_{i}^{g}$. The first 9 covariates are the same for all 5 tests (PEER factor 1 – PEER factor 7, age, sex). The tests also include a covariate and coefficient for treatment type ($x_{treat}, \beta_{treat}$), symptom severity at baseline ($x_{MA BL}$, $\beta_{MA BL}$), and treatment response ($x_{TR},\beta_{TR}$). In test 3 Wald tests are used to test if the difference between placebo and vortioxetine ($\beta_{treat}$) is equal to zero for each gene. In test 4 and 5 Wald tests are used to test if treatment response ($\beta_{TR}$) is constant for each gene using baseline and week 8 samples, respectively. Thus, the models for test 3, 4 and 5 using DESeq2 are:

$Test 3: {log2 q}_{g}= \sum_{i=1}^{9} x_{i}\beta_{i}^{g}+\boldsymbol{x}_{\boldsymbol{treat}}\boldsymbol{\beta}_{\boldsymbol{treat}}^{\boldsymbol{g}}$

$Test 4 and 5: {log2 q}_{g}=\sum_{i=1}^{9} x_{i}\beta_{i}^{g}+x_{treat}\beta_{treat}^{g}+x_{MA BL}\beta_{MA BL}^{g}+\boldsymbol{x}_{\boldsymbol{TR}}\boldsymbol{\beta}_{\boldsymbol{TR}}^{\boldsymbol{g}}$

where $K_{g}\sim NB\left( \mu_{g},a_{g} \right)$and $\mu_{g}= s_{g}q_{g}$

DESeq2’s function Shrinkage was used to shrink the log2FC towards 0 for genes with low information, such as high variance and low counts. The shrunken log2FC are reported in the results for these analyses.

**Genes differentially expressed between vortioxetine and placebo**

Comparing patients treated with vortioxetine to patients treated with placebo at week 8 identified no significant genes but 8 genes had a p-value < 0.001, see Supplementary Material 2 Table 1.

**Gene expression related to treatment response at baseline or week 8**

Change in gene expression related to treatment response was investigated at baseline and at week 8 for both treatment types (placebo and vortioxetine), see Supplementary Material 2 Table 3 and 4. Genes with an FDR < 0.05 are presented in Table 1. It was also explored if the significant changes in gene expression were associated with depression symptom severity at baseline. Furthermore, it was explored if the relationship between treatment response and the significant changes in gene expression depended on the type of treatment (placebo or vortioxetine) by including an interaction term between treatment response and type of treatment in the model.

At baseline, gene expression of 12 genes predicted treatment response with a p-value < 0.001 out of which 1 were significant. Downregulation of *PROK2* (FDR = 0.0016) significantly predicted a poorer treatment response regardless of treatment type. At week 8, 22 genes with a p-value < 0.001 had a gene expression related to treatment response but no genes were significant.

***Table 1.*** *Genes with differential expression correlated to treatment response at baseline.*

| **Gene** | **Mean** | **Log2FC (SE)** | **FDR** | **PVE (%)** | **P-value MADRS BL** | **P-value treatment** |
| --- | --- | --- | --- | --- | --- | --- |
| **Baseline** | | | | | | |
| ***PROK2*** | 859.53* | -0.0081 (0.0014) | 0.0016 | 3.32 | 0.89 | 0.60 |
| ***SOCS3*** | 435.35* | -0.0071 (0.0014) | 0.047 | 4.16 | 0.37 | 0.57 |
| ***GNG10*** | 295.27* | -0.0064 (0.0014) | 0.047 | 0.78 | 0.44 | 0.54 |
| **Week 8** | | | | | | |
| ***SOCS3*** | 414.30* | -0.0074 *(*0.0015*)* | 0.021 | 4.10 | 0.66 | 0.039 |
| **Note:** Mean with * = mean of normalized counts divided by size factor obtained using DESeq2; Mean without * = mean of counts per million using DREAM; Log2FC (SE) = log2 fold change (standard error); FDR = BH adjusted p-value; PVE = Proportion of Variance in gene expression Explained by treatment response; P-value MADRS BL = P-value of association between gene expression and depression symptom severity at baseline; P-value treatment = p-value of the interaction effect between treatment response and treatment type. | | | | | | |

**C: Choosing method for gene expression analysis**

Various methods can be used to conduct gene expression analyses in R. Among the methods are Limma [1], DREAM [2], and DESeq2 [3]. The methods are based on different normalization methods, regression models, and test statistics, see Table 2. DREAM is built as an extension to Limma and should be applied when conducting analyses with repeated measures. When there are no repeated measures DREAM and Limma perform identical analyses. We investigated which of these methods were best for our dataset. As a basis for comparison, basic gene expression tests were conducted in order to evaluate the power of the chosen methods and their false positive rates. DEseq2 is based on count normalization while the other methods are based on quantile quantile (QQ) normalization.

***Table 2****. Methods to conduct gene expression analyses.*

|  | **Normalization** | **Regression model** | **Test statistics** |
| --- | --- | --- | --- |
| **DREAM** | Trimmed Mean of M values | Mixed model based on normal distribution | Moderated t-statistics using an empirical Bayes approach testing whether the corresponding regression coefficient is different from 0 |
| **Limma** | Trimmed Mean of M values | Linear model based on normal distribution | t-statistic for testing whether the corresponding regression coefficient is different from 0 |
| **DESeq2** | DESeq2’s median of ratios | generalized linear model based negative binomial distribution | Wald statistic testing whether the corresponding regression coefficient is different from 0 |
| **Basic Linear model** | QQ normalization | Linear model based on normal distribution | t-statistic for testing whether the corresponding regression coefficient is different from 0 |
|  | | | |

We evaluated the methods by investigating the distribution of p-values when performing gene expression analyses using only the baseline samples. We wanted the method that had the greatest power which is reflected in significant p-values when the null hypothesis is false. However, when the null hypothesis is true we wanted a method that did not show signs of inflation of the p-value distribution.

First, we explored inflation in the p-value distribution for differentially expressed genes between placebo and vortioxetine at baseline. Since the patients had not received any treatment at baseline, then the null hypothesis was true, and we expected the p-values to follow a uniform distribution which can be evaluated in a QQ plot. There were no repeated measures in this analysis, therefore Limma and DREAM performed this analysis similarly. The QQ plots generated for the simple linear model followed the diagonal line in the QQ plots (see Figure 4A), whereas the QQ plots generated by limma/DREAM and DESeq2 were more conservative. The QQ plot for DESeq2 is visualized in Figure 4B. None of the tests showed inflation in the p-value distribution or generated false positives.


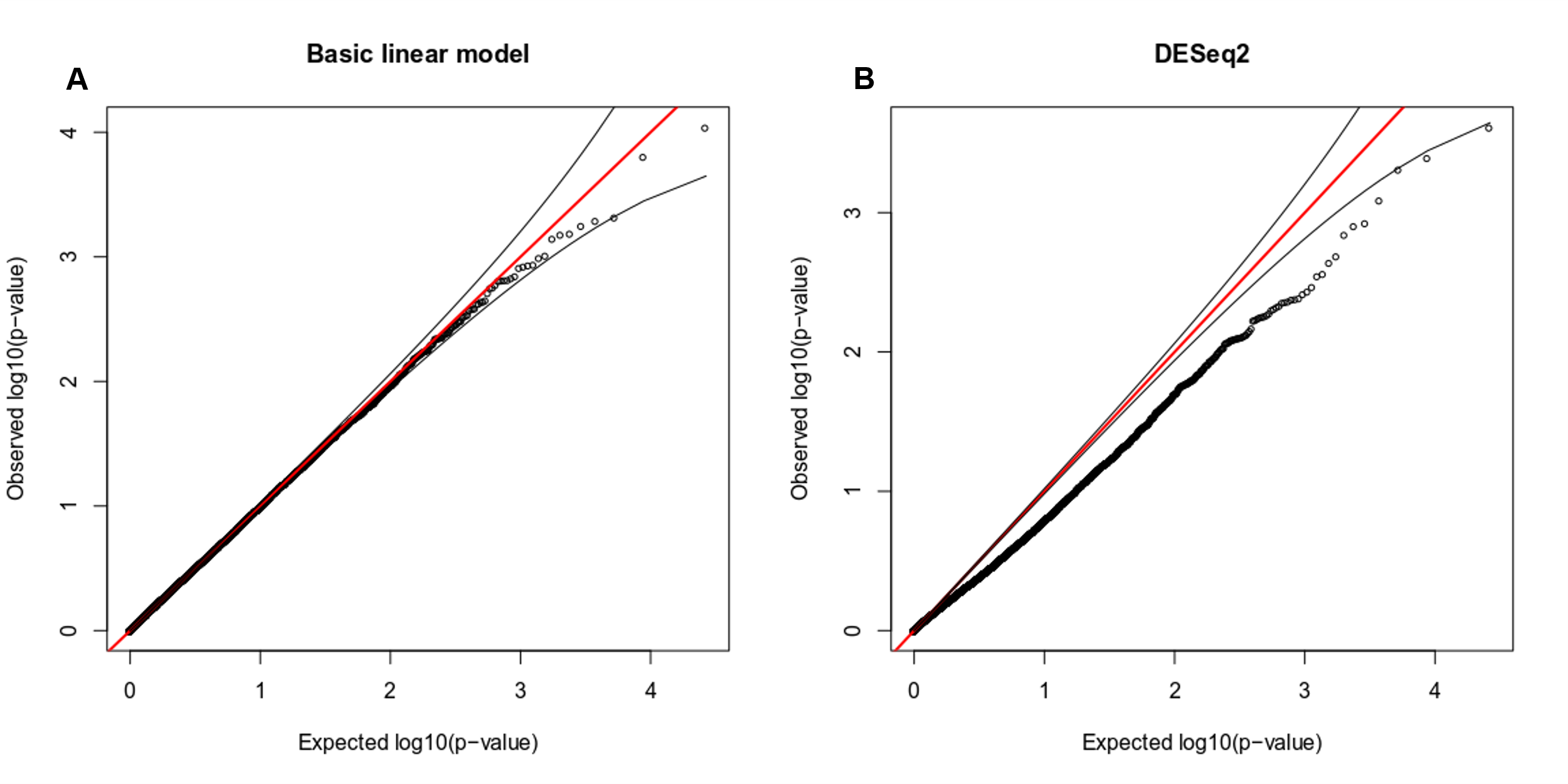


*Figure 4. QQ plots for gene expression between placebo and vortioxetine at baseline.* ***A*** *QQ plot based on p-values generated using a basic linear model.* ***B*** *QQ plot based on p-values generated using DESeq2.*

In order to determine which of the methods had the most power, we investigated differential expression between males and females. In this gene expression test we expected many strongly differentially expressed genes between the two groups. Thus in this test we expect the null hypothesis to be false for a very large number of the genes. Therefore in this test we were looking for the methods that generated the most significant p-values. We summarize the results using: The p-value of the most significantly differentially expressed gene, the number of highly significantly differentially expressed genes, and the number of norminally significantly differentially expressed genes.

***Table 3****. Variables describing the p-value distribution of gene expression between males and females
at baseline generated using methods for conducting gene expression analysis.*

| **Method** | **Most significant p-value** | **# P-values < 0.05** | **# P-values < 10^-15^** |
| --- | --- | --- | --- |
| **Basic linear model** | 1.726e-28 | 1370 | 5 |
| **DESeq2** | 3.252e-96 | 1789 | 21 |
| **Limma/DREAM** | 9.132e-43 | 1814 | 14 |
|  | | | |

Deseq2 showed the highest power when investigating differentially expressed genes between males and females using the baseline samples (see Table 3 and Figure 5.B) and Limma/DREAM had the second-best. However, the disadvantage of DESeq2 is that this method is less suitable for paired analysis. Consequently, we applied DESeq2 for unpaired gene expression analyses and we used individual random effects in DREAM when including both time points in the analysis.

**D: PEER factors**

Identification and removal of unknown sources of variation in gene expression, such as multi-site effects and batch effects, are strongly recommend and can improve RNA-seq studies significantly [4]. In this study we used PEER factors [5]. A study evaluating different methods used to remove variation in RNA-seq data showed, that PEER factors [5] removed the majority of false positives, were best at removing site-specific bias and removed variance significantly associated with GC-content, gene body coverage uniformity, and average base error rate and insert size. [4] PEER uses a Bayesian approach to calculate hidden factors, called PEER factors, explaining as much of the expression variability as possible. Subsequently, the PEER factors can be used as covariates in differential gene expression analysis. PEER factors do not have to be orthogonal (like principal components), and they can be calculated taking known covariates into account [5].

In the current study, the PEER factors were calculated using all samples and accounting for all covariates used in the experimental design. First, 20 PEER factors were generated. Next, a suitable number of PEER factors to include in the experimental design had to be determined. Including PEER factors which remove unknown sources of variation as covariates in a regression model can reduce noise and false positives and increase power to detect significant genes. However, adding too many covariates in a regression analysis can result in reduced power. Therefore, it was investigated how a different number of PEER factors affected the p-value distribution of gene expression between males and females at baseline using DESeq2, see Table 4. Besides sex and PEER factors, age was the only other covariate in these tests.

Including PEER factors improved the power of the tests. The p-value of the gene (KDM5C) with the most significant p-value was decreased from 3.252e-96 to 4.1191e-150 just by including the first 3 PEER factors. Including more than 7 PEER factors as covariates reduced the power of the gene expression tests. Based on the distribution of p-values, the most suitable number of PEER factors appeared to be 4 or 7. Next, we examined the distribution of variance across all genes when a different number of covariates were included in the experimental design. When no PEER factors were included in the experimental design the median variance explained by the residuals was 91.19 %. Adding 4 PEER factors to the experimental design captured a total median of 27.59 % of the variability genome-wide and the median variance explained by the residuals was reduced to 48.11 %. Including 7 PEER factors captured a total median of 32.26 % of the variability genome-wide and the median variance explained by the residuals was reduced to 34.32 %. Since, including 7 PEER factors as covariates showed reasonable p-value distribution and explained the second highest amount of variance genome-wide, this number of PEER factors were chosen.

***Table 4****. Variables describing the p-value distribution and variance of gene expression between males and females at baseline generated using DESeq2 and a different number of PEER factors.*

| **No. of PEER factors** | **Highest p-value** | **No. p-values < 0.05** | **No. p-values < 10*10^-15^** | **PVE PEER factors (%)** | **PVE residuals (%)** |
| --- | --- | --- | --- | --- | --- |
| 0 | 3.252e-96 | 1789 | 21 | 0 | 91.19 |
| 1 | 1.6553e-96 | 2361 | 29 | 7.82 | 77.19 |
| 2 | 4.8986e-107 | 2426 | 37 | 18.59 | 60.61 |
| 3 | 4.1191e-150 | 2517 | 49 | 28.22 | 48.57 |
| 4 | 1.2996e-151 | 2608 | 48 | 27.59 | 48.11 |
| 5 | 2.5155e-148 | 2053 | 40 | 31.28 | 41.47 |
| 6 | 1.4631e-146 | 1990 | 37 | 32.73 | 38.79 |
| 7 | 2.7625e-146 | 2224 | 53 | 32.26 | 34.32 |
| 8 | 6.3333e-133 | 1950 | 50 | 31.78 | 33.69 |
| PVE: Proportion of Variance in gene expression | | | | | |


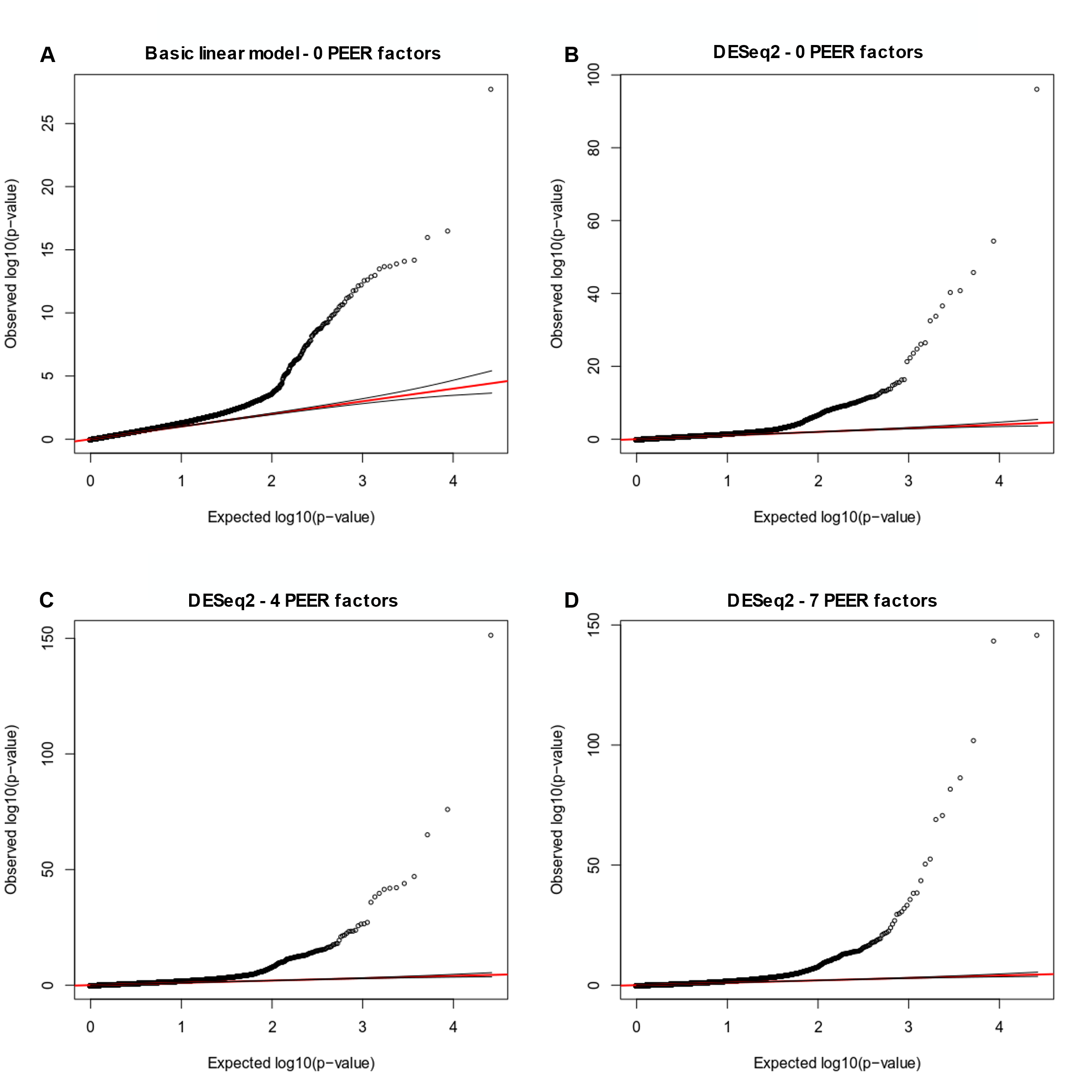


*Figure 5. QQ plots for gene expression between males and females at baseline.* ***A*** *QQ plot based on p-values generated using a basic linear model with age as the only covariate.* ***B*** *QQ plot based on p-values generated using DESeq2 with age as the only covariate.* ***C*** *QQ plot based on p-values generated using DESeq2 with age and 4 PEER factors as covariates.* ***D*** *QQ plot based on p-values generated using DESeq2 with age and 7 PEER factors as covariates.*

**REFERENCES**

1. Ritchie ME, Phipson B, Wu D, Hu Y, Law CW, Shi W, et al. Limma powers differential expression analyses for RNA-sequencing and microarray studies. Nucleic Acids Res. 2015. https://doi.org/10.1093/nar/gkv007.

2. Hoffman GE, Roussos P. dream: Powerful differential expression analysis for repeated measures designs. BioRxiv. 2018. https://doi.org/10.1101/432567.

3. Love MI, Huber W, Anders S. Moderated estimation of fold change and dispersion for RNA-seq data with DESeq2. Genome Biol. 2014. https://doi.org/10.1186/s13059-014-0550-8.

4. Li S, Labaj PP, Zumbo P, Sykacek P, Shi W, Shi L, et al. Detecting and correcting systematic variation in large-scale RNA sequencing data. Nat Biotechnol. 2014. https://doi.org/10.1038/nbt.3000.

5. Stegle O, Parts L, Piipari M, Winn J, Durbin R. Using probabilistic estimation of expression residuals (PEER) to obtain increased power and interpretability of gene expression analyses. Nat Protoc. 2012. https://doi.org/10.1038/nprot.2011.457.

***Table 5.*** *Demographics and baseline assessments.*

| **Characteristics** | **Placebo (N=97)**  Mean(SD)/% | **Vortioxetine 10 mg (N=92)**  Mean(SD)/% | **Vortioxetine 20 mg (N=92)**  Mean(SD)/% | **Vortioxetine (N=184)**  Mean(SD)/% |
| --- | --- | --- | --- | --- |
| **Demographics** |  |  |  |  |
| Gender (male) | 38.14 % | 35.87 % | 31.52 % | 33.70 % |
| Age (yr) | 44.69 (12.37) | 46 (12.45) | 44.65 (11.15) | (45.33) |
| Length of current MDE (wk) |  |  |  |  |
| Previous MDEs (N) | 3.07 (1.44) | 3.23 (1.64) | 3.32 (1.99) | 3.27 (1.82) |
| **BL assessment scores** |  |  |  |  |
| MADRS total score | 31.56 (4.11) | 31.75 (3.78) | 32.39 (3.81) | 32.07 (3.80) |
| DSST correct symbols | 43.31 (15.10) | 41.36 (13.46) | 41.77 (11.98) | 41.57(12.72) |
| **Note:** MDE =Major Depressive Episode; MADRS = Montgomery–Åsberg Depression Rating Scale; DSST = Digit Symbol Substitution Test. | | | | |

**
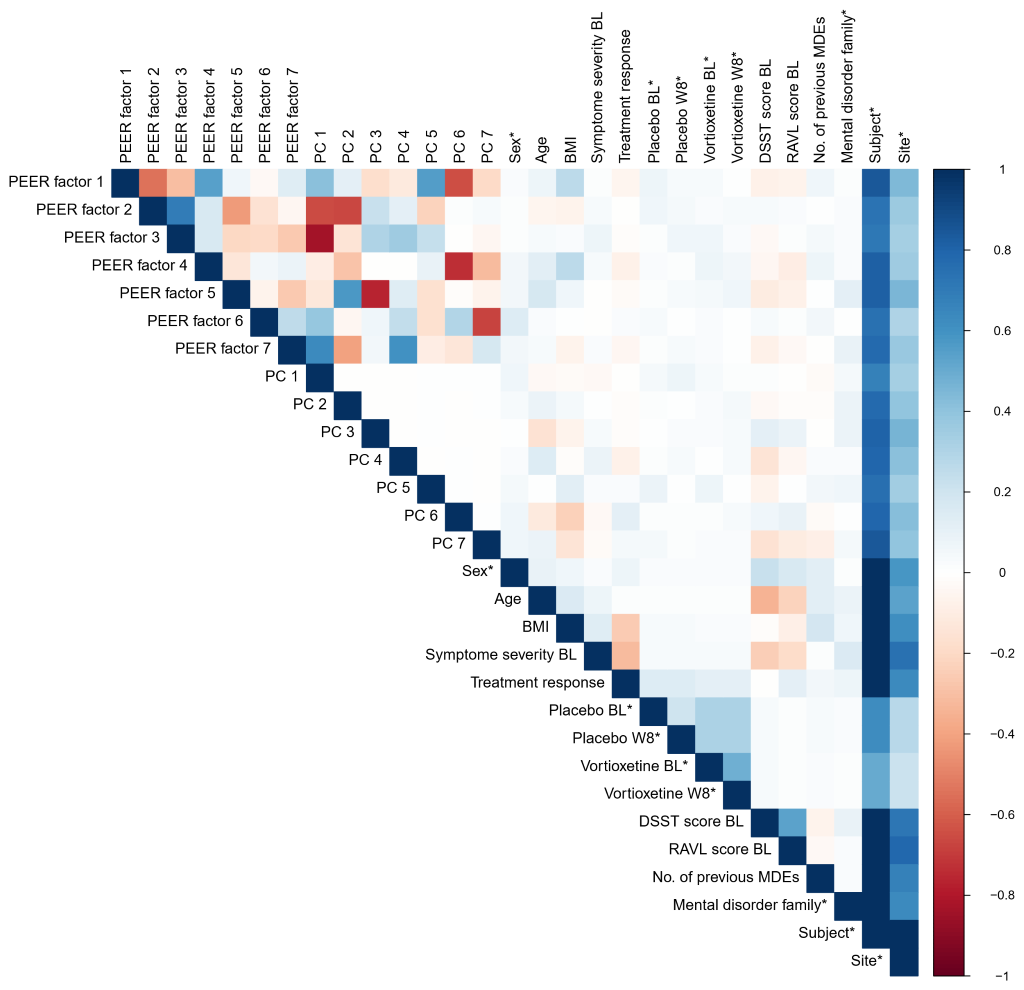
**

**Figure 6**. Heat map of Spearman correlation for pairs of variables assessing how much each pair co-vary from -1 to 1. Correlations with a p-value < 0.05 are considered insignificant and have the color white. * indicate categorical variables. For the categorical variables are canonical correlation for pairs of variables applied assessing how much they co-vary from 0 to 1.
